# Dissolution Behavior of Fluoroalkylated Diazonaphthoquinone and Its Blends with Fluorinated Copolymers under UV Irradiation

**DOI:** 10.3390/molecules28196784

**Published:** 2023-09-24

**Authors:** Gayoung Kim, Sae-Eun Kang, Doo Hong Kim, Jong-In Won, Yejin Ku, Jongchan Son, Jin-Kyun Lee, Byung Jun Jung

**Affiliations:** 1 Program in Environment and Polymer Engineering, Inha University, Incheon 22212, Republic of Korea; brrxzz@naver.com (G.K.); dkdlehdcns22@naver.com (Y.K.); 2 Department of Polymer Science and Engineering, Inha University, Incheon 22212, Republic of Korea; rkdtodms0216@naver.com (S.-E.K.); no2608@naver.com (J.-I.W.); thswhdcks1@naver.com (J.S.); 3 Department of Materials Science and Engineering, University of Seoul, Seoul 02504, Republic of Korea; danny4578@uos.ac.kr

**Keywords:** diazonaphthoquinone (DNQ), fluoropolymer, photolithography, photoresist, organic light-emitting diode (OLED)

## Abstract

This article reports on the synthesis of materials containing both a fluoroalkyl group and a diazonaphthoquinone (DNQ) moiety as well as the fabrication of negative- and positive-tone stencil patterns. Additionally, the photoreaction mechanism that contributes to the pattern formation process is discussed, and the application of these materials is explored in the pixel-formation process in organic light-emitting diode (OLED) displays. Fluoroalkylated diazonaphthoquinone (**R_F_2D1**) was synthesized using chemically binding a DNQ unit, which can be converted into carboxylic acid derivatives having stronger polarity, with two fluorinated alkyl chains. The purified compound is found to be soluble in a nonpolar fluorous solvent and can be uniformly coated as a thin film. When the thin film of **R_F_2D1** is exposed to 365 nm UV light, its solubility in a fluorous solvent decreases due to the Wolff rearrangement and subsequent hydrolysis of a ketene moiety. In contrast, when a mixture of **R_F_2D1** and a hydrophobic, fluorinated copolymer is tested for the patterning process, the copolymer delays the conversion of the ketene intermediate to carboxylic acid, resulting in the dissolution of the exposed areas in the fluorous solvent. Finally, the applicability of these materials in micropatterning is demonstrated by adopting them in the *orthogonal* photolithography process to create pixels of OLEDs.

## 1. Introduction

The demand for display devices that offer high resolution, large area, and the ability to be transformed into various shapes has increased. To meet these demands, there have been continuous innovations in electronic materials and manufacturing technologies, which have enabled the commercialization of liquid crystal, plasma panel, and organic light-emitting diode (OLED) displays. Among them, OLED-based displays have now achieved a firm commercial position based on their excellent viewing angle, brightness, contrast ratio, and light weight [1,2,3]. Currently, OLED technology, beyond its traditional applications such as televisions or mobile phones, is advancing towards the visualization of augmented reality (AR) and virtual reality (VR) [4,5].

At present, the only commercially adopted method for manufacturing OLED pixels is thermal vacuum deposition using a fine metal mask (FMM). While it simultaneously achieves an emitting film coating and pixel patterning, the fabrication of large-area display panels with a high enough resolution below 10 μm is limited due to the sagging phenomenon of the substrate and FMM under gravity [6,7,8]. Various alternative methods have been explored to overcome this problem, including inkjet printing [9,10,11], laser-induced thermal imaging [12,13], and micro-contact printing [14,15], but their commercialization has been constrained due to technological limitations. As another option, the utilization of photolithographic patterning technology for OLED pixel formation has been proposed, which enables high-resolution patterning with large substrates and features facile registration capabilities [16,17,18,19,20]. It is however acknowledged that the charge transporting and light-emitting layers of OLEDs can be physically and chemically damaged by the solvents commonly used for photoresist casting, development, and stripping, and its application for OLED pixel patterning has not been regarded seriously.

To address these issues, we have conducted research on patterning organic semiconducting materials, relying on a modified photolithographic process that uses highly fluorinated or fluorous solvents. In general, these fluorous molecules have chemical orthogonality with organic semiconducting materials [21,22,23]. To proceed in this way, it is crucial to acquire a photosensitive material or photoresist with sufficient solubility in those media to construct stencils on top of an organic semiconducting layer. Until recently, negative-tone fluorinated photoresists that leave the UV-exposed domains as stencil patterns have predominantly been employed [24]. Based on simple and intuitive chemical mechanisms, including increase in molecular polarity via deprotection reactions or crosslinking among the photoresist polymer chains under UV irradiation, they lose solubility in nonpolar fluorous developing solvents forming negative-tone images.

These working mechanisms however cause hassles in the stripping process, which involves removing the photoresist stencils that are no longer needed in the following steps [25]. Due to the photoresist’s reduced solubility, dissolving it using mild fluorous solvents is not straightforward. Often, harsh processing conditions become necessary, such as using stronger solvents or applying mechanical forces in the form of ultrasonication, which can potentially damage the patterned OLED pixels. Recognizing this limitation, we have been persistently striving to prepare positive-tone fluorinated photoresists that show increased solubility when exposed to UV light [26]. Recently, we reported a positive-tone material based on the photoisomerization chemistry of spiropyran moieties whose solubility increases in nonpolar fluorinated solvents [27].

This success urged us to explore other chemical possibilities for positive-tone fluorinated photoresists, and we paid attention to the conventional diazonaphthoquinone (DNQ)-containing materials employed for i-line (365 nm UV light) photolithography. DNQ-based photoresists are a type of nonchemically amplified photoresists that do not depend on an acid-catalyzed deprotection reaction for solubility change. Generally, they consist of a mixture of DNQ derivatives and a novolak resin, a phenol-formaldehyde condensation polymer [28,29,30,31,32,33,34]. Novolak resin contributes to the formation of rigid photoresist films that are washed away in an aqueous base solution because it has acidic phenolic hydroxyl groups. The DNQ derivative does not have particularly acidic functions and thus acts as a dissolution inhibitor when it is mixed with novolak resin. Therefore, before UV irradiation, the mixed film of the DNQ derivative and novolak resin does not dissolve appreciably in an aqueous basic developer solution.

The DNQ units in the photoresist film undergo the Wolff rearrangement reaction upon UV irradiation, resulting in the formation of highly reactive ketene functional groups. These reactive units then react with moisture inside the film or in the atmosphere to generate carboxylic acid functional groups. Due to this chemical change in the DNQ derivatives, the dissolution rate of the exposed regions of the photoresist film increases abruptly in an aqueous base solution. Hence, the mixture of the DNQ derivative and novolak works as a positive-tone patterning material in conventional i-line photolithographic processes.

DNQ photoresists have been widely used in the semiconductor and display industries due to their several advantages, including excellent sensitivity, low cost, and commercial availability. Furthermore, they have been employed in a variety of other futuristic applications, such as producing amphiphilic polymer assemblies for controlled release [35] and preparing materials for holograms recording with pulsed lasers [36]. Therefore, we were motivated to examine the capabilities of DNQ-based photoresists in a photolithographic system operated with fluorous solvents. In this study, we investigated the solubility-changing behavior of a fluoroalkylated DNQ derivative in fluorous solvents in the UV irradiation step. We also explored its potential use as a stencil-building material in *orthogonal* photolithography for OLED pixel patterning.

## 2. Results and Discussion

### 2.1. Synthesis of Materials

**Scheme 1:**
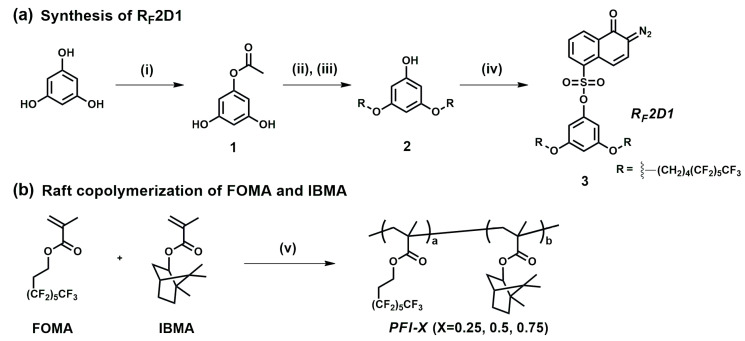
Synthesis of (**a**) **R_F_2D1** and (**b**) fluoroalkylated polymers (**PFI-X**). *Reagents and conditions*: (i) Ac_2_O, pyridine, 120 °C, 3 h; (ii) 1,1,1,2,2,3,3,4,4,5,5,6,6-tridecafluoro-10-iododecane, K_2_CO_3_, DMF, 70 °C, 5 h; (iii) KOH (1.0 M in DI water), EtOH, 80 °C, 1 h; (iv) 5-DNQ-Cl, Et_3_N, THF, r.t., 2 h; (v) 4-cyano-4-[(dodecylsulfanylthiocarbonyl)sulfanyl]pentanoic acid (CDSTSP), α,α′-azobis(isobutyronitrile) (AIBN), THF, benzotrifluoride (BTF), 80 °C, 12 h.

In this study, a photoreactive compound was designed using a strategy of combining fluoroalkyl chains with nonfluorinated DNQ units to achieve a fluorous-solvent-soluble photoresist. It was also desired to create a structure with low crystallinity to use the product in photolithography. To accomplish this, two fluoroalkyl chains and a DNQ unit were introduced into phloroglucinol, which possesses a rigid aromatic core and three hydroxyl groups. The compound difluoroalkylated diazonaphthoquinone (**R_F_2D1**, **3**) was synthesized in four steps (Scheme 1a). First, acetylation was performed at one of the three reaction sites of phloroglucinol, resulting in the synthesis of monoacetylphloroglucinol (**1**) with a yield of 38%. Subsequently, disubstituted compounds were synthesized via performing nucleophilic substitution on 1, 1, 1, 2, 2, 3, 3, 4, 4, 5, 5, 6, 6-tridecafluoro-10-iododecane under basic conditions. Following this, difluoroalkylated phenol (**2**) was obtained with a yield of 36% through deacetylation under basic conditions. The ^1^H, ^13^C, and ^19^F NMR spectra for these compounds are presented in Figures S1–S4. Finally, **R_F_2D1** (**3**) was prepared with a yield of 67% by reacting the phenol **2** with 5-DNQ-Cl. Confirmation of the synthesized material was accomplished through ^1^H-NMR measurement, where peaks ranging from 6.0 to 8.8 ppm corresponding to the DNQ moiety and peaks ranging from 1.6 to 3.9 ppm corresponding to the hydrogens in the fluorinated alkyl unit were observed (Figure S5). The ^13^C and ^19^F NMR measurements also supported this structural identification of **R_F_2D1** (Figures S6 and S7). When analyzed using high-performance liquid chromatography (HPLC), **R_F_2D1** exhibited purity of 94.7% (Figure S8). Additionally, the ESI-TOF-MS measurement confirmed the synthesis (Figure S9).

Fluoroalkylated copolymers were synthesized to observe any changes in solubility and patterning properties via mixing with **R_F_2D1**. These fluorinated polymers were obtained by copolymerizing 1*H*,1*H*,2*H*,2*H*-perfluorooctyl methacrylate (FOMA) and isobornyl methacrylate (IBMA) (Scheme 1b). The logic behind this selection was to achieve resins with both high solubility in fluorous solvents and a suitable glass transition temperature (*T*_g_). Additionally, copolymers with a low polydispersity index (PDI) were desired and could be achieved using a polymerization protocol known as reversible addition-fragmentation chain-transfer (RAFT) polymerization. To control the glass transition temperature and related pattering characteristics, the polymerization process was conducted with different ratios of IBMA comonomer, resulting in the synthesis of three copolymers (**PFI-X**, X = 0.25, 0.5, 0.75). The mole ratios between the comonomers in all copolymers were checked using ^1^H-NMR analysis (Figures S10–S12). The molar compositions of IBMA to FOMA in **PFI-0.25**, **PFI-0.5**, and **PFI-0.75** were 0.15, 0.38, and 0.67, respectively (Table 1). The copolymers exhibited number average molecular weights (*M_n_*) ranging from approximately 8100 to 8200 g/mol and relatively narrow PDIs ranging from 1.1 to 1.3 (Table 1). *T*_g_ data were obtained from differential scanning calorimetry (DSC) measurements. As the molar composition of IBMA increased, the *T*_g_ of the copolymers also increased to 28, 34, and 40 °C. Detailed information regarding the monomer feed ratio, composition, *M_n_*, PDI, and *T*_g_ for **PFI-X** (X = 0.25, 0.5, 0.75) is presented in Table 1 and is summarized in Figures S13–S16.

Among the three copolymers, **PFI-0.5** showed favorable solubility in fluorous solvents and was thus identified as a suitable photoresist candidate for pattern formation.

### 2.2. Photolithographic Evaluation

**Scheme 2:**
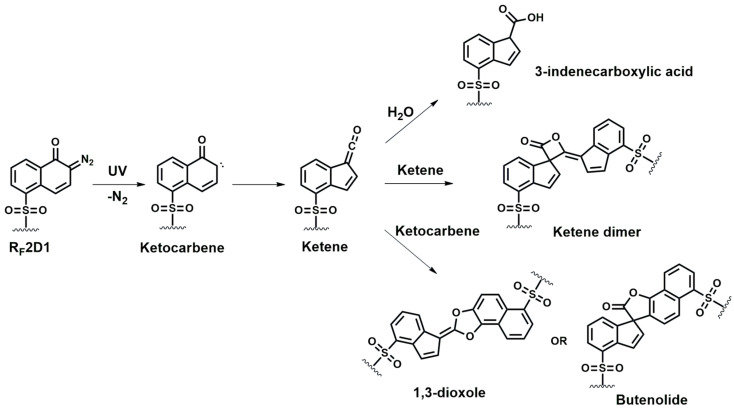
Plausible photoreaction pathways of **R_F_2D1**.

**Figure 1:**
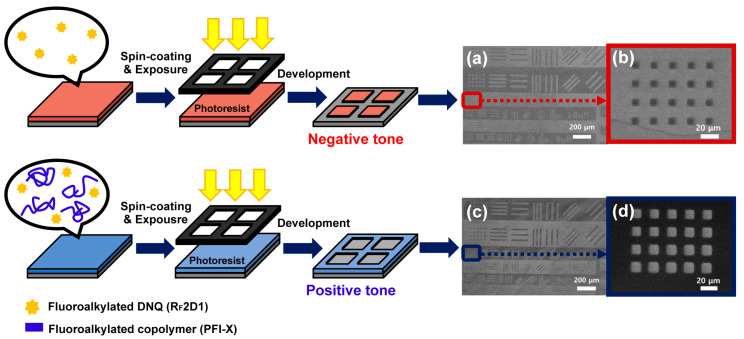
Photolithographic patterning process and scanning electron microscope (SEM) images of the developed photoresist patterns. SEM images of the films: (**a**) **R_F_2D1** (negative tone) and (**c**) mixture of **PFI-0.5** and **R_F_2D1** (positive tone). The magnified pictures in (**b**) and (**d**) are SEM images of 10 μm-sized patterns.

To evaluate the photopatterning capabilities of the fluoroalkylated derivative **R_F_2D1**, an experiment was conducted using a 365 nm light source. Initially, a 10%(*w*/*v*) solution of **R_F_2D1** in a fluorous solvent (PF-7600) was spin-coated on a Si wafer, and the wafer was heated at 70 °C for 60 s (soft bake). The resulting thin film was then exposed to UV light with an energy dosage of 0.16 J/cm^2^. Subsequently, the film was developed using a mixture of two fluorous solvents, FC-770 and FC-3283 in a 1:10 ratio. This development step resulted in the formation of a negative-tone pattern, in which the UV-exposed areas exhibited reduced solubility (Figure 1a,b and Figure S17). It was hypothesized that upon UV irradiation, a Wolff rearrangement reaction of **R_F_2D1** would occur, followed by the generation of a ketene intermediate (Scheme 2). This reactive derivative would react with the moisture inside the film and be converted into a carboxylic acid moiety with strong polarity. We anticipated that this transformation would decrease the solubility of **R_F_2D1** in nonpolar fluorous developing media.

Along with the patterning evaluation with **R_F_2D1** only, another experiment was conducted with a mixture of **R_F_2D1** and fluoroalkylated copolymer. **PFI-0.5** was blended with **R_F_2D1** at a weight ratio of 5:1, and the mixture was dissolved in HFE-7500 to form a 15 wt% coating solution. It was spin-coated on a Si substrate, and the resulting thin film was soft-baked. After UV exposure with an energy dosage of 0.12 J/cm^2^, a development process was carried out with a mixture of HFE-7300 and FC-770 in a 1:10 ratio. As a result, the solubility of the exposed areas increased; thus, those regions were washed off to form a positive-tone image (Figure 1c,d and Figure S17). The same experiments were performed with **PFI-0.25** and **PFI-0.75**, and the results are summarized in Figure S17. The chemical structures of the fluorous solvents employed in this study are shown in Figure S18.

### 2.3. Mechanism Study

#### 2.3.1. FT-IR Spectra

**Figure 2:**
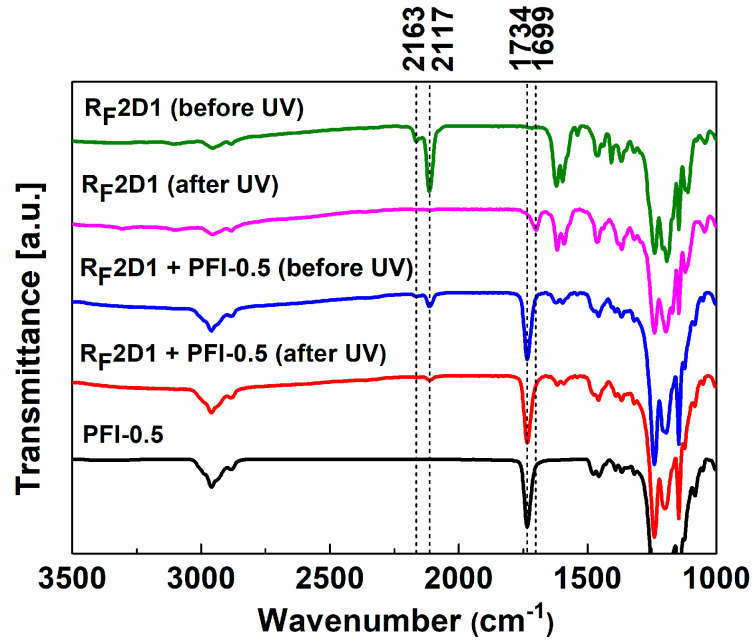
FT-IR spectra of films cast with **R_F_2D1** and mixture of **PFI-0.5** and **R_F_2D1** before and after UV exposure.

To confirm the chemical structural changes in **R_F_2D1** before and after UV exposure, a thin film of the compound was prepared and subjected to FT-IR measurements (Figure 2). A comparison of the spectra before and after exposure revealed differences. After UV exposure, the absorption bands corresponding to the diazo functional group (N≡N) at 2166 and 2120 cm^−1^ disappeared. Furthermore, a strong absorption band at 1697 cm^−1^ corresponding to the carbonyl moiety (C=O) of carboxylic acid emerged [37,38]. Additionally, a band attributed to the carboxyl group (COOH) was also observed in the 2500–3500 cm^−1^ region. Even before exposure, a band associated with OH was detected in the 2500–3500 cm^−1^ region, which probably originated from the moisture within the film. The resulting reactive ketene intermediate could then be converted into indenecarboxylic acid through its reaction with the moisture present in the thin film or the surrounding air.

FT-IR measurements were also conducted on the film containing both the polymer **PFI-0.5** and **R_F_2D1** before and after UV exposure. Here, it was observed that the absorption bands corresponding to the diazo functional group (N≡N) at 2166 and 2120 cm^−1^ decreased after exposure. However, in this case, due to the overlap with the ester carbonyl peak (1734 cm^−1^) of the copolymer, it was challenging to confirm the formation of the carbonyl bond (C=O) from **R_F_2D1**.

#### 2.3.2. Influence of Moisture in Films

**Figure 3:**
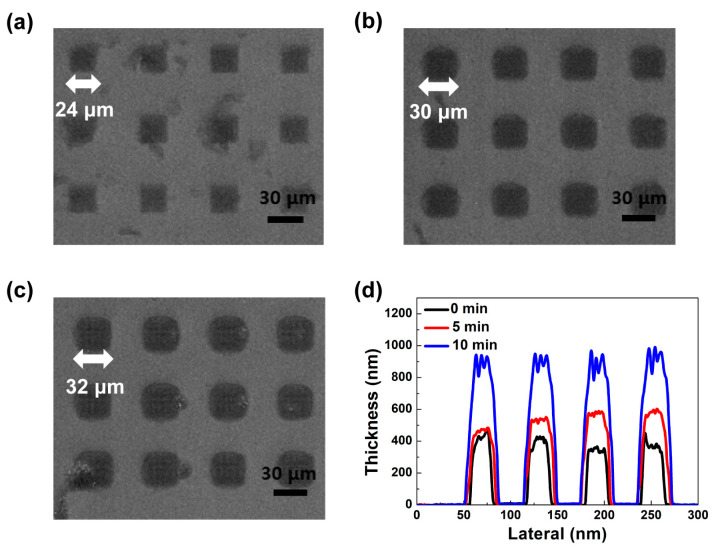
SEM images of 30 μm size negative-tone patterns using **R_F_2D1**: (**a**) development immediately after UV exposure (pattern size: 24 μm); (**b**) development 5 min after UV exposure (pattern size: 30 μm); and (**c**) development 10 min after UV exposure (pattern size: 32 μm). (**d**) Film thickness of 30 μm patterns (development at 0, 5, and 10 min after UV exposure).

Additional experiments were performed to investigate the influence of moisture on the solubility of **R_F_2D1** (Figure 3). This experiment was conducted at a humidity of 67% and a temperature of 26 °C. A solution of **R_F_2D1** dissolved in PF-7600 at a concentration of 15% (*w*/*v*) was spin-coated on a Si substrate, followed by soft-baking. The coated film was exposed to UV light at an energy of 0.24 J/cm^2^. Variations in the thickness and size of the material’s patterns were observed after development. Here, we inserted delay time, 0, 5, and 10 min, between the UV exposure and the pattern development step. The results indicated that as the delay time increased, the thickness of the remaining patterns also increased, accompanied by an increase in the width of the remaining negative-tone patterns. This observation was attributed to the gradual conversion of the exposed **R_F_2D1** molecules into carboxylic acid derivatives in the presence of moisture. This chemical conversion resulted in the reduced solubility of the exposed regions and an increase in the proportion of insoluble residual matter, forming thicker and wider negative-tone patterns.

Based on the patterning results achieved with **R_F_2D1**, an explanation of the positive-tone pattern formation presented in Section 2.2. is proposed. It is highly likely that the copolymer **PFI-X** prohibits and delays the reaction of **R_F_2D1** with moisture, and thus the unreacted ketene intermediate or its dimerization product acts as a dissolution promoter in a film composed of **PFI-X** and **R_F_2D1** (Scheme 2) [39]. Consequently, the solubility of the exposed regions increases in nonpolar fluorous solvents, and positive-tone images are left in the blended films.

### 2.4. Fabrication and Performance Evaluation of OLED Pixels

**Figure 4:**
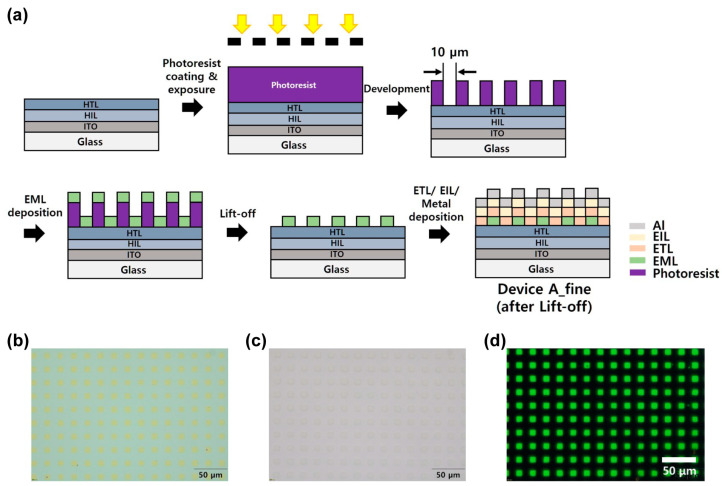
(**a**) Process flow using a photomask containing 10 μm × 10 μm checkerboard patterns for fine patterning of OLED layer. Optical microscope images of Device A: (**b**) after lift-off process, (**c**) after metal deposition, and (**d**) during emission under an electric bias.

To validate the applicability of the positive-tone fluorinated patterning material (a mixture of **R_F_2D1** and **PFI-0.5**) for OLED pixel patterning, an experiment of tailoring an OLED thin film into micropixels was carried out (Figure 4a). The process involved the sequential deposition of a hole-injecting layer (HIL) and hole-transporting layers (HTLs) on a 25 mm × 25 mm glass substrate containing a 2 mm × 2 mm ITO electrode. A photoresist solution containing **R_F_2D1** and **PFI-0.5** was dissolved in HFE-7500 in a ratio of 1:5 and then spin-coated onto the substrate, followed by soft-baking at 80 °C for 60 s. Then, 365 nm UV light was irradiated through a photomask with a checkerboard pattern of 10 μm × 10 μm holes. A development process was performed using a mixed solvent of HFE-7300 and FC-770 in a 1:10 ratio. As a result, certain parts of the HTLs could be exposed to the external environment that were not covered with the photoresist film. Emitting layers (EMLs) were deposited onto the patterned organic film stack. A lift-off process followed to remove of the photoresist film and the EMLs lying on it by immersing the substrate in HFE-7300. Finally, additional layers including an electron-transporting layer (ETL), electron-injecting layer (EIL), and Al were deposited sequentially to complete the fabrication of a pixel-patterned OLED device. The chemical structures of the OLED materials employed in this study are shown in Figure S19. We refer to this device as Device A. The optical microscope images of Device A after the lift-off process, after encapsulation, and during emission under electric bias are presented in Figure 4b–d. These results confirmed that micropatterned OLED devices were successfully fabricated using a photolithography protocol employing a positive-tone photoresist material.

**Figure 5:**
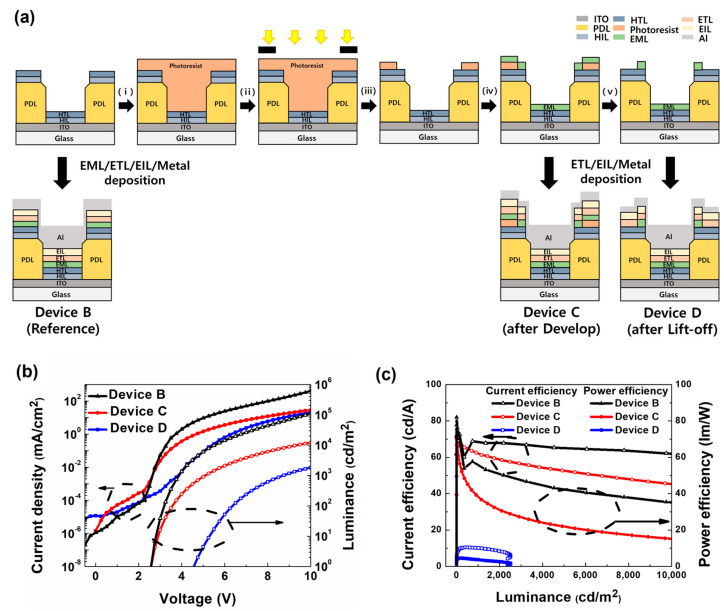
(**a**) Process flow using a photomask containing 3.5 mm × 3.5 mm electrode patterns for OLED characterization: (i) spin-coating, (ii) exposure, (iii) development, (iv) EML deposition, and (v) lift-off. (**b**) Current density–voltage–luminance characteristics; (**c**) current efficiency–luminance characteristics of reference device (Device B) and processed device (Devices C and D).

After demonstrating the applicability of the photolithographic patterning protocol for OLED pixel patterning, the performance of the patterned devices was investigated. Three devices (Devices B, C, and D) were manufactured for comparison, as depicted in Figure 5a. Device B did not undergo the photopatterning process at all. Device C did not undergo the lift-off process, and Device D underwent the lift-off step. A photo of the device obtained through the photolithography process is presented in Figure S20. Comparing the current density–voltage-–luminance (J-V-L) relationships of the three devices, it was observed that the turn-on voltage increased in Device D, which underwent the lift-off process. This performance deterioration was attributed to physical damage to the surface of the EML during the lift-off process and chemical damage caused by exposure to air. Device C exhibited the same turn-on voltage as the reference Device B, though the photolithography process involving photoresist coating and development was performed on the HTL (Figure 5b). However, the current efficiency of Device C was slightly lower than that of Device B when emitting light with high luminance (Figure 5c). Although these results indicated that an OLED device could be fabricated using the photolithography process with fluorinated materials, further efforts would be required to enhance the device performance to a satisfactory level.

## 3. Experimental Section

### 3.1. Materials

Phloroglucinol (≥99%), isobornyl methacrylate (IBMA, technical grade), tetrahydrofuran (THF, anhydrous, ≥99.9%), benzotrifluoride (BTF, anhydrous), N,N-dimethylformamide (DMF, ≥99.8%, anhydrous), and 1,3,5-tri(m-pyridin-3-ylphenyl)benzene (TmPyPB, 98%) were purchased from Sigma-Aldrich. 1*H*,1*H*,2*H*,2*H*-Perfluorooctyl methacrylate (FOMA) was procured from U-Chem, Republic of Korea. IBMA and FOMA were used after being passed through a short plug of aluminum oxide. Anhydrous K_2_CO_3_ and α,α′-azobis(isobutyronitrile) (AIBN, ≥98.0%) were purchased from Junsei Chemical, Japan. AIBN was used after recrystallization from a mixture of MeOH and CHCl_3_. Ac_2_O (99%) was obtained from Samchun Chemicals, Republic of Korea. Pyridine (≥99%) and Et_3_N (≥99%) were purchased from Thermo Scientific. 1,2-Naphthoquinone-2-diazide-5-sulfonyl chloride (5-DNQ-Cl) was donated by Miwon Chemical Co., Republic of Korea. 1,1,1,2,2,3,3,4,4,5,5,6,6-Tridecafluoro-10-iododecane and 4-cyano-4-[(dodecylsulfanylthiocarbonyl)sulfanyl]pentanoic acid (CDSTSP) were synthesized according to a method in the literature [40,41]. 1,4,5,8,9,11-Hexaazatriphenylene hexacarbonitrile (HATCN, ≥99.9%), N,N′-di(1-naphthyl)-N,N′-diphenyl-(1,10-biphenyl)-4,4′-diamine (NPB, ≥99.9%), 2,2′,2′′-(1,3,5-benzinetriyl)-tris(1-phenyl-1-H-benzimidazole) (TPBi, ≥99.9%), and tris [2-phenylpyridine-C2,N]iridium (III) (Ir(ppy)_3_, ≥99.5%) were supplied by OSM, Republic of Korea. Tris(4-carbazoyl-9-ylphenyl)amine (TCTA) was procured from Luminescence Technology Co. (Lumtec), Taiwan. LiF (≥99.9%) and Al (≥99.99%) were purchased from Taewon Science Co. (iTASCO), Republic of Korea. 1,1,1,2,3,3-Hexafluoro-4-(1,1,2,3,3,3-hexafluoropropoxy)-pentane (PF-7600), 3-Ethoxy-1,1,1,2,3,4,4,5,5,6,6,6-dodecafluoro-2-(trifluoromethyl)-hexane (HFE-7500), 1,1,1,2,3,4,5,6,6,6-Decafluoro-3-methoxy-4-trifluoromethylpentane (HFE-7300), 1,1,1,2,2,-3,3,4,4,5,5,6,6,7,7,8,8,9,9,10,10,10-Docosafluorodecane (FC-770), and 1,1,2,2,3,3,3-heptafluoro-N,N-bis(1,1,2,2,3,3,3-hepta-fluoropropyl)propan-1-amine (FC-3283) were obtained from 3M Korea.

### 3.2. Characterization

The ^1^H-NMR and ^13^C NMR spectra of the synthesized materials were recorded on a Bruker Advance III spectrometer (400 MHz for ^1^H NMR, 101 MHz for ^13^C NMR and 376 MHz for ^19^F NMR) using the chemical shift of residual aprotic solvents (acetone at δ = 2.05 ppm for ^1^H NMR, CDCl_3_ at δ = 7.26 ppm for ^1^H NMR and at δ = 77.16 ppm for ^13^C NMR) as a reference. Herein, all the chemical shifts are quoted in parts per million (ppm) relative to the internal reference, and the coupling constants (*J*) are reported in Hz. The multiplicity of the signal is indicated as follows: s (singlet), d (doublet), t (triplet), dd (doublet of doublets), and m (multiplet). Fourier transform infrared (FT-IR) spectroscopy was performed on a Bruker VERTEX 80V with film samples on double-sided polished wafers. A spot-type UV-LED (365 nm; UV SMT Technology) and an MJB4 mask aligner (Hg light source and deep UV cut-off filter; SUSS Micro Tec. This equipment is located at the 3D Convergence Center of Inha University.) were utilized as the UV exposure source. High-performance liquid chromatography (HPLC) was conducted on a Shimazu LC-20 system equipped with a UV/Vis detector by eluting isooctane and tert-butyl methyl ether. Differential scanning calorimetry (DSC) (Perkin Elmer DSC 6000) was employed to measure the glass transition temperatures (*T*_g_) of the polymer binders at a heating/cooling rate of 5 °C min^−^^1^ under a N_2_ atmosphere during the heating/cooling/heating cycles. Electrospray ionization time-of-flight mass spectra (ESI-TOF-MS) were recorded on a Bruker Q-TOF spectrometer. Size exclusion chromatography was performed on a Thermo Scientific Ultimate 3000 chromatography system with an eluent of THF at a flow rate of 1 mL/min at 35 °C. The chromatograms obtained with the UV/Vis detector (λ = 209 nm) were analyzed with a calibration curve constructed using ten standard polystyrene (PS) samples in a range of 1.22–2700 kg/mol. The thicknesses of the thin films on the Si wafer were measured using an Alpha-step D-300 Stylus Profiler. The surface treatment of the substrate was performed using a CUTE-1MPR (Femto Science), which is plasma equipment that uses the capacitively coupled plasma (CCP) method. The organic thin film was deposited using a DOVE-1500 vacuum thermal evaporator (DOV). The current density–voltage–luminance (J-V-L) characteristics were measured using a B2912A Source Measuring Unit (SMU) (Agilent Technology, Santa Clara, CA, USA) and a calibrated Si photodiode (Thorlabs, Newton, NJ, USA) according to a reported method [42].

### 3.3. Synthesis of Materials

#### 3.3.1. Synthesis of Fluorinated DNQ Derivative (**R_F_2D1**)

##### Monoacetylphloroglucinol (**1**)

A mixture of phloroglucinol (2.00 g, 15.9 mmol) and acetic anhydride (1.78 g, 17.5 mmol) was added to a 250 cm^3^ round-bottom flask at room temperature (25 °C). Subsequently, pyridine (20.0 cm^3^) was added as both a catalyst and a solvent to form a mixed solution, which was then stirred at 120 °C for 3 h and diluted with ethyl acetate (EtOAc) (80.0 cm^3^). The resulting solution was washed with 2.0 M HCl aqueous solution (100 cm^3^) to remove the pyridine. Subsequently, the organic layer was washed with water and brine. After drying the solution using anhydrous magnesium sulfate (MgSO_4_), the crude product was purified via column chromatography (Silica gel, CH_2_Cl_2_:EtOAc = 3:1). By concentrating the crude product under reduced pressure, monoacetylphloroglucinol (**1**) was obtained with a yield of 1.02 g (38%); ^1^H NMR (400 MHz, acetone-d6): *δ* = 8.40 (s, 2 H, 2 × Ar-O*H*), 6.24 (s, 1 H, Ar-*H*), 6.11 (s, 2 H, 2 × Ar-*H*), 2.19 (s, 3 H, C*H*_3_COO).

The δ_H_ data are in agreement with the literature values [43].

##### Difluoroalkylated Phenol (**2**)

Monoacetylphloroglucinol (1, 0.620 g, 3.71 mmol) and 1,1,1,2,2,3,3,4,4,5,5,6,6-tridecafluoro-10-iododecane (3.73 g, 7.42 mmol) were added to DMF (12.0 cm^3^). K_2_CO_3_ (1.03 g, 7.42 mmol) was then added to the mixed solution and stirred at 70 °C for 5 h. Subsequently, the solution was diluted by adding EtOAc (80.0 cm^3^), and the organic solvent layer was extracted by washing it with water and brine. After drying the solution using anhydrous MgSO_4_, the resulting crude product was concentrated under reduced pressure. This concentrated crude product was then dissolved in EtOH (5.00 cm^3^), after which 1.0 M KOH aqueous solution (5.00 cm^3^) was added. The mixture was then stirred at 80 °C for 1 h. Once the reaction was completed, the solution was cooled to room temperature and neutralized using 1.0 M HCl aqueous solution (10.0 cm^3^). After diluting with EtOAc (30.0 cm^3^), the organic solution was washed with water and brine three times. The crude product was then purified via column chromatography ((silica gel, EtOAc:hexane = 1:3). By concentrating the crude product under reduced pressure, difluoroalkylated phenol (**2**, 1.17 g, 36%) was obtained. The HPLC analysis indicated a purity of 95.5%; ^1^H NMR (400 MHz, CDCl_3_): *δ* = 6.05 (s, 1 H, Ar-*H*), 6.01 (s, 2 H, 2 × Ar-*H*), 4.66 (s, 1 H, Ar-O*H*), 3.95 (t, J = 5.5 Hz, 4 H, 2 × ArOC*H*_2_), 2.29–2.03 (m, 4 H, 2 × C*H*_2_CF_3_), 1.91–1.74 (m, 8 H, 4 × C*H*_2_). ^13^C NMR (101 MHz, CDCl_3_): *δ* = 161.35, 157.60, 122.34–120.90 (m, CH_2_*C*F_2_), 118.92 (q, *J*_CF_ = 31.5 Hz, *C*F_3_), 116.22 (t, *J*_CF_ = 32 Hz, *C*F_2_), 114.64–112.97 (m, *C*F_2_), 112.27–110.23 (m, *C*F_2_), 109.58–107.69 (m, *C*F_2_), 95.46, 94.94, 67.82, 30.88 (t, *J*_CF_ = 22.5 Hz, *C*H_2_CF_2_), 28.92, 17.44; ^19^F NMR (376 MHz, CDCl_3_): *δ* = −80.89 (t, *J*_FF_ = 10 Hz, 6F), −114.46 (m, 4F), −121.95 (m, 4F), −122.92 (m, 4F), −123.58 (m, 4F), −126.19 (m, 4F); m/z (ESI-TOF-MS) 897.0898 [(M + Na)^+^. C26H20F26NaO3 requires M, 897.0895].

##### Difluoroalkylated Diazonaphthoquinone (**R_F_2D1**, **3**)

Difluoroalkylated phenol (**2**, 1.17g, 1.34 mmol) and 5-DNQ-Cl (0.539 g, 2.01 mmol) were dissolved in 50.0 cm^3^ of THF. Et_3_N (0.271 g, 2.68 mmol) was then added to the mixed solution and stirred at room temperature for 2 h. The solution was then diluted with EtOAc (70.0 cm^3^), and the organic layer was extracted with water and brine. After drying over anhydrous MgSO_4_, the crude product was concentrated under reduced pressure. Difluoroalkylated diazonaphthoquinone (**3**, **R_F_2D1**, 2.21 g, 67%) was obtained as a yellow powder by diluting it with EtOAc and reprecipitating in cold MeOH (350 cm^3^). The HPLC analysis revealed a purity of 94.7%; ^1^H NMR (400 MHz, CDCl_3_): *δ* = 8.67 (d, J = 7.5 Hz, 1H, Ar-*H* in DNQ), 8.22 (dd, J = 7.5, 1 Hz, 1 H, Ar-*H* in DNQ), 7.55 (d, J = 10 Hz, 1H, Ar-*H* in DNQ) 7.50 (t, J = 7.5 Hz, 1H, Ar-*H* in DNQ), 7.22 (d, J = 10 Hz, 1 H, Ar-*H* in DNQ), 6.27 (t, J = 2 Hz, 1 H, Ar-*H*), 6.10 (d, J = 2 Hz, 2 H, 2 × Ar-*H*), 3.82 (t, J = 5 Hz, 4 H, 2 × ArOC*H*_2_), 2.20–2.04 (m, 4 H, 2 × C*H*_2_CF_3_), 1.84–1.70 (m, 8 H, 4 × C*H*_2_) ^13^C NMR (101 MHz, CDCl_3_): *δ* = 177.58, 160.48, 150.78, 135.36, 135.06, 132.16, 131.60, 131.09, 125.80, 121.96–120.59 (m, CH_2_*C*F_2_), 121.12, 118.59 (q, *J*_CF_ = 31.5 Hz, *C*F_3_), 115.88 (t, *J*_CF_ = 33.5 Hz, *C*F_2_), 114.29-112.62 (m, *C*F_2_), 112.44, 111.98–109.98 (m, *C*F_2_), 109.29–107.64 (m, *C*F_2_), 100.98, 100.31, 79.20, 67.60, 30.54 (t, *J*_CF_ = 22.5 Hz, *C*H_2_CF_2_), 28.39, 17.10. ^19^F NMR (376 MHz, CDCl_3_): *δ* = −80.82 (t, *J*_FF_ = 10 Hz, 6F), −114.41 (m, 4F), −121.91 (m, 4F), −122.87 (m, 4F), −123.53 (m, 4F), −126.13 (m, 4F); m/z (ESI-TOF-MS) 1129.0847 [(M + Na)^+^. C36H24F26N2NaO6S requires M, 1129.0838].

#### 3.3.2. Synthesis of Copolymer

##### Raft Copolymer of FOMA and IBMA (**PFI-X**)

FOMA (1.00 g, 2.31 mmol), IBMA (0.257 g, 1.16 mmol), THF (2.00 cm^3^), and BTF (2.00 cm^3^) were added to a 25 cm^3^ vial and underwent bubbling with N_2_ gas. Purified AIBN (6.30 mg, 0.0386 mmol) and CDSTSP (0.0311 g, 0.0771 mmol) were then placed in a 25 cm^3^ Schlenk flask, which was subsequently sealed and the atmosphere inside was replaced with N_2_. The solution of FOMA and IBMA was transferred to the Schlenk flask and subsequently degassed using three freeze–pump–thaw cycles. The degassed solution was stirred at 80 °C for 12 h. The resulting solution was added dropwise to 250 cm^3^ of MeOH, and the precipitate was recovered via filtration. The resulting precipitate was then dried under reduced pressure to obtain a pale-yellow powder polymer, **PFI-0.5** (1.10 g, 87%). The other copolymers with adjusted composition ratios of the monomers (**PFI-X**, X = 0.25, 0.75) were also synthesized using a similar method to that used for **FSI-0.5**. In this case, X represents the equivalent ratio of IBMA when the equivalent of FOMA is 1.

### 3.4. Lithographic Evaluation

#### 3.4.1. **R_F_2D1** Film

A 10% (*w*/*v*) solution of **R_F_2D1** in PF-7600 was spin-coated onto a silicon wafer (500 rpm, 60 s) followed by soft baking at 70 °C for 60 s. The resulting cast film was exposed to 365 nm UV light (0.16 J/cm^2^) using a photomask. Subsequently, the exposed **R_F_2D1** film was washed with a solvent mixture of FC-770: FC-3283 in a ratio of 1:10, resulting in the formation of a negative-tone pattern.

#### 3.4.2. Mixture Films of **R_F_2D1** + **PFI–X** (X = 0.25, 0.5, 0.75)

A 15% (*w*/*v*) solution of **R_F_2D1** and **PFI-X** (X = 0.25, 0.5, 0.75) at a ratio of 1:5 (*w*/*w*) in HFE-7500 was spin-coated onto a silicon wafer (500 rpm for 60 s), followed by soft-baking at 80 °C for 60 s. All three samples were then exposed to 365 nm UV light at a dosage of 0.12 J/cm^2^ using a photomask. The exposed **PFI-0.25**, **PFI-0.5**, and **PFI-0.75** films were washed with a mixture of FC-3283 and FC-770 in a 1:1 (*v*/*v*) ratio and HFE-7300 and FC-770 at ratios of 1:10 and 1:1, respectively. In all three cases, a positive-tone pattern was obtained.

### 3.5. Fabrication of Micropatterned OLED Pixels

The process was conducted using an ITO-glass substrate measuring 25 mm × 25 mm, with a pixel area of 2 mm × 2 mm separated by a pixel define layer (PDL). The substrate was first ultrasonically cleaned and dried in deionized (DI) water before undergoing surface treatment using plasma equipment. The surface treatment was performed at 100 W and 100 kHz at a partial pressure of O_2_ of 1.35 × 10^−^^1^ torr for 5 min. The treated substrate was then loaded into a vacuum thermal evaporator maintained at a pressure level of 6.0 × 10^−^^7^ torr. An organic thin film was deposited using the following materials, and the deposition rate and total thickness were monitored using a quartz crystal microbalance (QCM). The host and dopant were co-deposited using different thickness monitors appropriately positioned within the same chamber. A 5 nm layer of HATCN was utilized as the hole injection layer (HIL). The hole transport layer (HTL) consisted of a 40 nm layer of NPB and a TCTA layer of 10 nm thickness. The emissive layer consisted of a bilayer structure. The first layer comprised 10 nm of TCTA doped with 8 wt% of the phosphorescent dopant (Ir(ppy)_3_). The second layer, with a thickness of 10 nm, consisted of TPBi doped with 8 wt% of Ir(ppy)_3_. For the electron transport layer (ETL), TmPyPB was employed. The electron injection layer and cathode of the OLED device comprised a 0.7 nm thick layer of LiF and a 75 nm thick layer of Al, respectively. Following deposition, the OLED device was encapsulated in a nitrogen atmosphere using a 20 mm × 20 mm glass lid, calcium oxide desiccant, and UV-curable epoxy resin. To achieve EML patterning, a photolithography process was performed on the HTL.

## 4. Conclusions

In this study, fluorinated photoresists containing DNQ units were achieved that exhibited good solubility in fluorous solvents. When **R_F_2D1** was used alone in the patterning process, a negative-tone pattern formed via a Wolff rearrangement upon UV irradiation and subsequent hydrolysis of a reactive ketene intermediate. We conducted FT-IR measurements and observed peaks corresponding to a carboxylic acid group that provided evidence of the Wolff rearrangement reaction. Additionally, changes in the solubility of **R_F_2D1** films were observed by measuring the thickness and size of the material’s patterns by inserting a time delay in between UV exposure and development step. The results revealed that both the thickness and size of the patterns increased as the delay time increased. On the other hand, when **R_F_2D1** was mixed with fluorinated copolymers (**PFI-X**), the solubility of the exposed areas of the blended films increased in fluorous developing media, resulting in the formation of a positive-tone image. The presence of the hydrophobic copolymer matrix delayed the conversion of the ketene intermediate into a carboxylic acid derivatives, thus allowing the ketene derivative to act as a dissolution promotor in the photoresist film. By applying the fluorinated photoresist to OLED pixel patterning through the *orthogonal* photolithography protocol, 10 μm × 10 μm OLED pixel arrays were successfully fabricated. This achievement demonstrated the potential of our method in the fine patterning of organic semiconductor devices.

## Figures and Tables

**Table 1:** Feed ratio, molar composition, molecular weight, PDI, and *T*_g_ data of **PFI-X** copolymers.

PFI-X	FOMA:IBMA		*M_n_*^b^ (g/mol)	PDI ^b^	*T*_g_^c^ (°C)
	Feed Ratio	Composition ^a^			
**PFI-0.25**	1:0.25	1:0.15	8200	1.16	27
**PFI-0.5**	1:0.5	1:0.38	8100	1.26	33
**PFI-0.75**	1:0.75	1:0.67	8100	1.29	42

^a^ Measured using NMR, ^b^ measured using gel permeation chromatography (GPC), ^c^ measured using DSC.

## Data Availability

Data can be found in the published manuscript and its Supplementary Materials.

## Supplementary Materials

The following supporting information can be downloaded at: https://www.mdpi.com/article/10.3390/molecules28196784/s1, Figure S1: ^1^H-NMR spectrum of monoacetylphloroglucinol; Figure S2: ^1^H-NMR spectrum of difluoroalkylated phenol; Figure S3: ^13^C-NMR spectrum of difluoroalkylated phenol; Figure S4: ^19^F-NMR spectrum of difluoroalkylated phenol; Figure S5: ^1^H-NMR spectrum of **R_F_2D1**; Figure S6: ^13^C-NMR spectrum of **R_F_2D1**; Figure S7: ^19^F-NMR spectrum of **R_F_2D1**; Figure S8: HPLC chromatograms of (a) difluoroalkylated phenol and (b) **R_F_2D1**; Figure S9: ESI-TOF-MS spectrum of **R_F_2D1**; Figure S10: ^1^H-NMR spectrum of **PFI-0.25**; Figure S11: ^1^H-NMR spectrum of **PFI-0.5**; Figure S12: ^1^H-NMR spectrum of **PFI-0.75**; Figure S13: GPC chromatogram of **PFI-0.25**; Figure S14: GPC chromatogram of **PFI-0.5**; Figure S15: GPC chromatogram of **PFI-0.75**; Figure S16: DSC curves of **PFI-X** (X=0.25, 0.5, 0.75); Figure S17: Optical microscope images of the films: (a) **R_F_2D1**, mixture of **R_F_2D1** and (b) **PFI-0.25**, (c) **PFI-0.5**, and (d) **PFI-0.75**; Figure S18: Chemical structures of process solvents employed in this study: (a) HFE-7300, (b) HFE-7500, (c) HFE-7600, (d) FC-770, and (e) FC-3283; Figure S19: Chemical structures of OLED materials; (a) HATCN, (b) NPB, (c) TCTA, (d) TPBi, (e) Ir(ppy)_3_ and (f) TmPyPB; Figure S20: Photographic images of processed device.

## Sample Availability

Samples of the compounds are available for the purpose of collaboration from the authors.

## References

[1] Boher P., Leroux T., Collomb-Patton V., Bignon T. (2015). Optical characterization of OLED displays. J. Soc. Inf. Disp..

[2] Geffroy B., Le Roy P., Prat C. (2006). Organic light-emitting diode (OLED) technology: Materials, devices and display technologies. Polym. Int..

[3] Yang H.I., Naveen K.R., Cho S.M., Kim J.Y., Jung Y.H., Kwon J.H. (2023). Systematic investigation on polymer layer selection for flexible thin film encapsulation. Org. Electron..

[4] Lee H.S., Jang S., Noh J., Jeon H., Choi B.-S., Jeon Y.M., Song K., Song J., Chu H.Y., Kim S. (2017). In 28-4L: Late-News Paper: An Ultra High Density 1.96” UHD 2250ppi Display. SID Symp. Dig. Tech. Pap..

[5] Ghosh A., Donoghue E.P., Khayrullin I., Ali T., Wacyk L., Tice K., Vazan F., Prache O., Wang Q., Sziklas L. (2017). 18-1: Invited Paper: Ultra-High-Brightness 2K x 2K Full-Color OLED Microdisplay Using Direct Patterning of OLED Emitters. SID Symp. Dig. Tech. Pap..

[6] Lih J.J., Chao C.I., Lee C.C. (2007). Novel pixel design for high-resolution AMOLED displays with a shadow mask. J. Soc. Inf. Disp..

[7] Kwon J.H. (2013). RGB color patterning for AMOLED TVs. Inf. Disp..

[8] Shin H.Y., Suh M.C. (2014). A study on full color organic light emitting diodes with blue common layer under the patterned emission layer. Org. Electron..

[9] Haskal E., Buechel M., Dijksman J., Duineveld P., Meulenkamp E., Mutsaers C., Sempel A., Snijder P., Vulto S., Van de Weijer P. (2002). 21.1: Ink Jet Printing of Passive-Matrix Polymer Light Emitting Displays. SID Symp. Dig. Tech. Pap..

[10] Hu Z., Yin Y., Ali M.U., Peng W., Zhang S., Li D., Zou T., Li Y., Jiao S., Chen S.-J. (2020). Inkjet printed uniform quantum dots as color conversion layers for full-color OLED displays. Nanoscale.

[11] Kang J.-g., Koo Y., Ha J., Lee C. (2020). 41-2: Invited Paper: Recent Developments in Inkjet-printed OLEDs for High Resolution, Large Area Applications. SID Symp. Dig. Tech. Pap..

[12] Lee S.T., Chin B.D., Kim M.H., Kang T.M., Song M.W., Lee J.H., Kim H.D., Chung H.K., Wolk M.B., Bellmann E. (2004). 29.3: A Novel Patterning Method for Full-Color Organic Light-Emitting Devices: Laser Induced Thermal Imaging (LITI). SID Symp. Dig. Tech. Pap..

[13] Suh M.C., Chin B.D., Kim M.H., Kang T.M., Lee S.T. (2003). Enhanced luminance of blue light-emitting polymers by blending with hole-transporting materials. Adv. Mater..

[14] Takakuwa A., Misaki M., Yoshida Y., Yase K. (2009). Micropatterning of emitting layers by microcontact printing and application to organic light-emitting diodes. Thin Solid. Film..

[15] Li J., Xu L., Tang C.W., Shestopalov A.A. (2016). High-resolution organic light-emitting diodes patterned via contact printing. ACS Appl. Mater. Interfaces.

[16] Gather M.C., Koehnen A., Falcou A., Becker H., Meerholz K. (2007). Solution-processed full-color polymer organic light-emitting diode displays fabricated by direct photolithography. Adv. Funct. Mater..

[17] Balocco C., Majewski L., Song A. (2006). Non-destructive patterning of conducting-polymer devices using subtractive photolithography. Org. Electron..

[18] Huang J., Xia R., Kim Y., Wang X., Dane J., Hofmann O., Mosley A., De Mello A., De Mello J., Bradley D. (2007). Patterning of organic devices by interlayer lithography. J. Mater. Chem..

[19] Jang W., Lee M., Kweon H., Park H.W., Yang J., Kim S., Jo H., Lee C., Cho J.H., Kwak K. (2021). Tetrabranched photo-crosslinker enables micrometer-scale patterning of light-emitting super yellow for high-resolution OLEDs. ACS Photonics.

[20] Yamamoto H., Kozawa T., Tagawa S. (2014). Study on dissolution behavior of polymer-bound and polymer-blended photo acid generator (PAG) resists by using quartz crystal microbalance (QCM) method. Microelectron. Eng..

[21] Zakhidov A.A., Lee J.K., Fong H.H., DeFranco J.A., Chatzichristidi M., Taylor P.G., Ober C.K., Malliaras G.G. (2008). Hydrofluoroethers as orthogonal solvents for the chemical processing of organic electronic materials. Adv. Mater..

[22] Choi Y.M., Shin H.Y., Son J., Park C., Park K.-W., Lee J.-K., Jung B.J. (2020). Two-color pixel patterning for high-resolution organic light-emitting displays using photolithography. Micromachines.

[23] Krotkus S., Ventsch F., Kasemann D., Zakhidov A.A., Hofmann S., Leo K., Gather M.C. (2014). Photo-patterning of highly efficient state-of-the-art phosphorescent OLEDs using orthogonal hydrofluoroethers. Adv. Opt. Mater..

[24] Son J., Oh H.-T., Kwon O.J., Lim J.-M., Jung H., Jung B.J., Hwang D.-H., Lee C., Lee J.-K., Yoon J.G. (2017). Highly soluble fluorous alkyl ether-tagged imaging materials for the photo-patterning of organic light-emitting devices. J. Mater. Chem. C.

[25] Son J., Shin H.Y., Choi Y.M., Chae S.G., Park C., Jung B.J., Lee J.-K. (2020). Descumming fluorous solution for photolithographic patterning of organic light-emitting diodes. Microelectron. Eng..

[26] Son J., Roh H., Shin H.Y., Park K.-W., Park C., Park H., Lee C., Kwak J., Jung B.J., Lee J.-K. (2020). Photo-cleavable perfluoroalkylated copolymers for tailoring quantum dot thin films. Polym. Chem..

[27] Park K.-W., Choi S., Choi Y.M., Kim G., Ku Y., Son J., Lee J.-K., Jung B.J., Kim M. (2023). Photoinduced Solubility Modulation in the Copolymers of Fluoroalkyl, Spiropyranyl, and Isobornyl Methacrylates. ACS Appl. Polym. Mater..

[28] Reiser A., Huang J., He X., Yeh T., Jha S., Shih H., Kim M., Han Y., Yan K. (2002). The molecular mechanism of novolak–diazonaphthoquinone resists. Eur. Polym. J..

[29] Nakayama T., Ueda M. (1999). A new positive-type photoresist based on mono-substituted hydroquinone calix [8] arene and diazonaphthoquinone. J. Mater. Chem..

[30] Andraos J., Chiang Y., Huang C., Kresge A., Scaiano J. (1993). Flash photolytic generation and study of ketene and carboxylic acid enol intermediates formed by the photolysis of diazonaphthoquinones in aqueous solution. J. Am. Chem. Soc..

[31] Vleggaar J., Huizer A., Kraakman P., Nijssen W., Visser R., Varma C. (1994). Photoinduced Wolff-rearrangement of 2-diazo-1-naphthoquinones: Evidence for the participation of a carbene intermediate. J. Am. Chem. Soc..

[32] Suwa M., Kamogawa M., Fujii M., Nishiyama T., Kozuka H. (2021). Intermolecular interactions between sol–gel-derived random-structure oligomeric silsesquioxanes and a diazonaphthoquinone derivative. Jpn. J. Appl. Phys..

[33] Wallraff G., Hinsberg W.D. (1999). Lithographic imaging techniques for the formation of nanoscopic features. Chem. Rev..

[34] Furuta A., Hanabata M. (1989). Novolak structures suitable for high resolution positive photoresists and application. J. Photopolym. Sci. Technol..

[35] Li Q., Cao Z., Wang G. (2018). Diazonaphthoquinone-based amphiphilic polymer assemblies for NIR/UV light-and pH-responsive controlled release. Polym. Chem..

[36] Zacharovas S.J., Bakanas R., Adlienė D., Šeperys R., Narmontas P., Jankauskaitė V. (2014). Holograms recording with pulsed laser on diazonaphthoquinone-novolac-based photoresists and their nanocomposites. Opt. Eng..

[37] Bratton D., Ayothi R., Deng H., Cao H.B., Ober C.K. (2007). Diazonaphthoquinone molecular glass photoresists: Patterning without chemical amplification. Chem. Mater..

[38] Wolpert D., Schade M., Brixner T. (2008). Femtosecond midinfrared study of the photoinduced Wolff rearrangement of diazonaphthoquinone. J. Chem. Phys..

[39] Omote T., Mochizuki H., Koseki K.I., Yamaoka T. (1990). Fluorine-containing photoreactive polyimide. 7. Photochemical reaction of pendant 1, 2-naphthoquinone diazide moieties in novel photoreactive polyimides. Macromolecules.

[40] Alvey L.J., Meier R., Soós T., Bernatis P., Gladysz J.A. (2000). Syntheses and carbonyliridium complexes of unsymmetrically substituted fluorous trialkylphosphanes: Precision tuning of electronic properties, including insulation of the perfluoroalkyl groups. Eur. J. Inorg. Chem..

[41] Moad G., Chong Y., Postma A., Rizzardo E., Thang S.H. (2005). Advances in RAFT polymerization: The synthesis of polymers with defined end-groups. Polymer.

[42] Forrest S.R., Bradley D.D., Thompson M.E. (2003). Measuring the efficiency of organic light-emitting devices. Adv. Mater..

[43] Baker M.B., Ferreira R.B., Tasseroul J., Lampkins A.J., Al Abbas A., Abboud K.A., Castellano R.K. (2016). Selective and sequential aminolysis of benzotrifuranone: Synergism of electronic effects and ring strain gradient. J. Org. Chem..

