# Supplementary material for: Dissolution Behavior of Fluoroalkylated Diazonaphthoquinone and Its Blends with Fluorinated Copolymers under UV Irradiation
Source: Molecules. 2023 Sep 24;28(19):6784. doi: 10.3390/molecules28196784 (PMC10574127; doi:10.3390/molecules28196784)
Supplement: Supplementary file 1 [file molecules-28-06784-s001.zip › molecules-2616135-supplementary.pdf]

Supporting Information

Dissolution behavior of fluoroalkylated diazonaphthoquinone and its blends with fluorinated copolymers under UV irradiation

Gayoung Kim[1], Sae-Eun Kang[2], Doo Hong Kim[3], Jong-In Won[2], Yejin Ku[1], Jongchan Son[2], Jin-Kyun Lee[1,2,*], Byung Jun Jung[3,*]

[1] Program in Environment and Polymer Engineering, Inha University, Incheon 22212, Republic of Korea

[2] Department of Polymer Science and Engineering, Inha University, Incheon 22212, Republic of Korea

[3] Department of Materials Science and Engineering, University of Seoul, Seoul 02504, Republic of Korea

*Correspondence: jkl36@inha.ac.kr and jungbj@uos.ac.kr

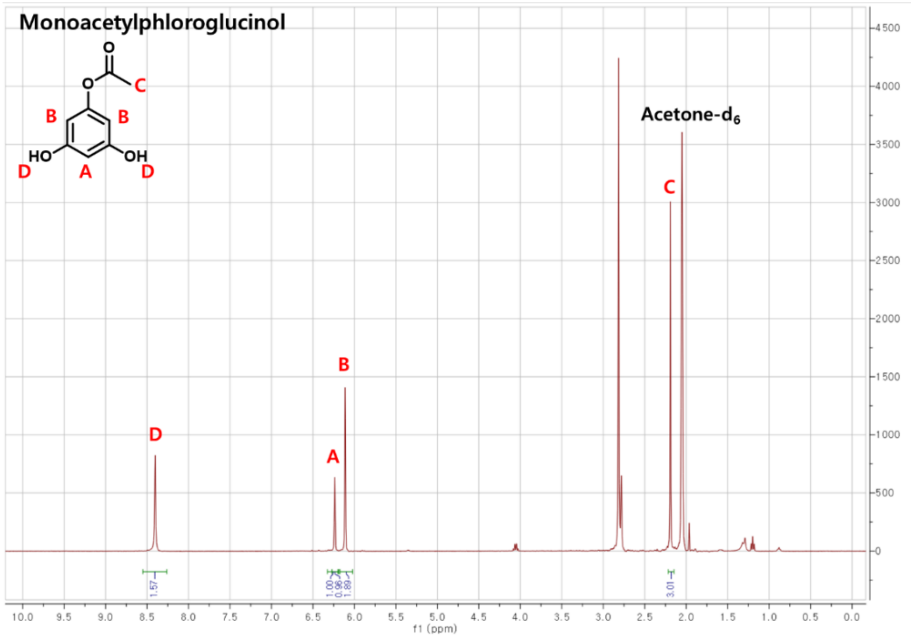

Figure S1. ^{1}H-NMR spectrum of monoacetylphloroglucinol.

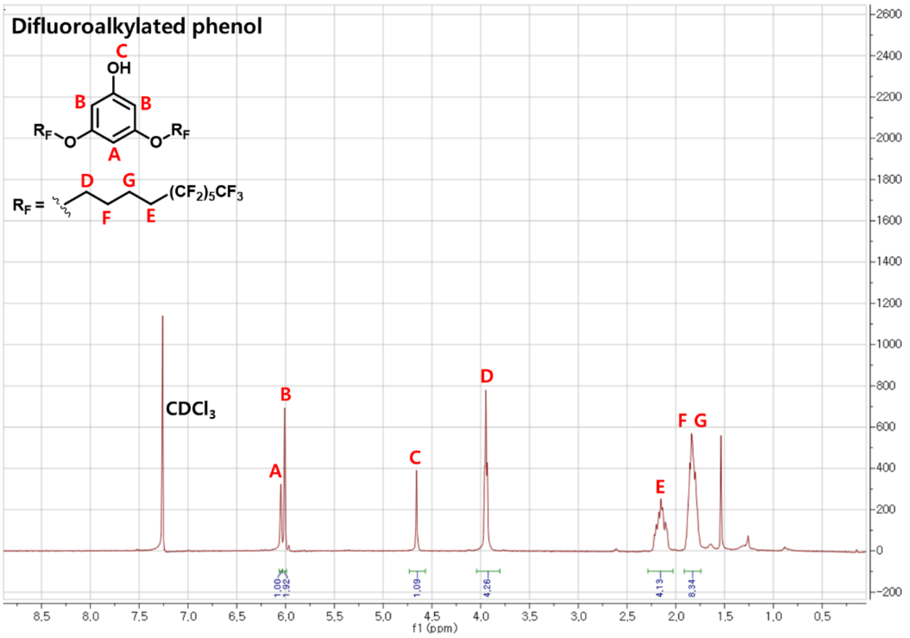

Figure S2. ^{1}H-NMR spectrum of difluoroalkylated phenol.

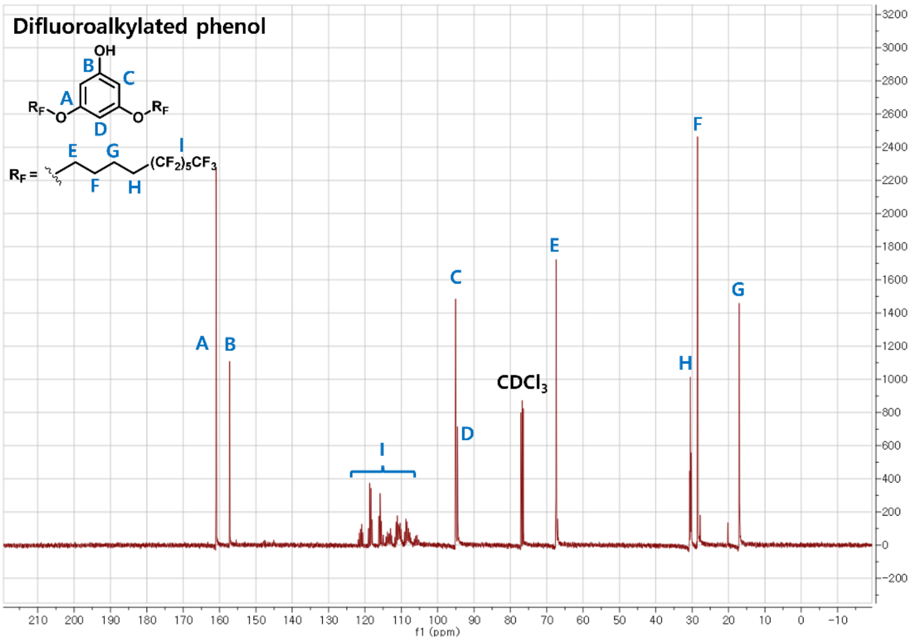

Figure S3. ^{13}C-NMR spectrum of difluoroalkylated phenol.

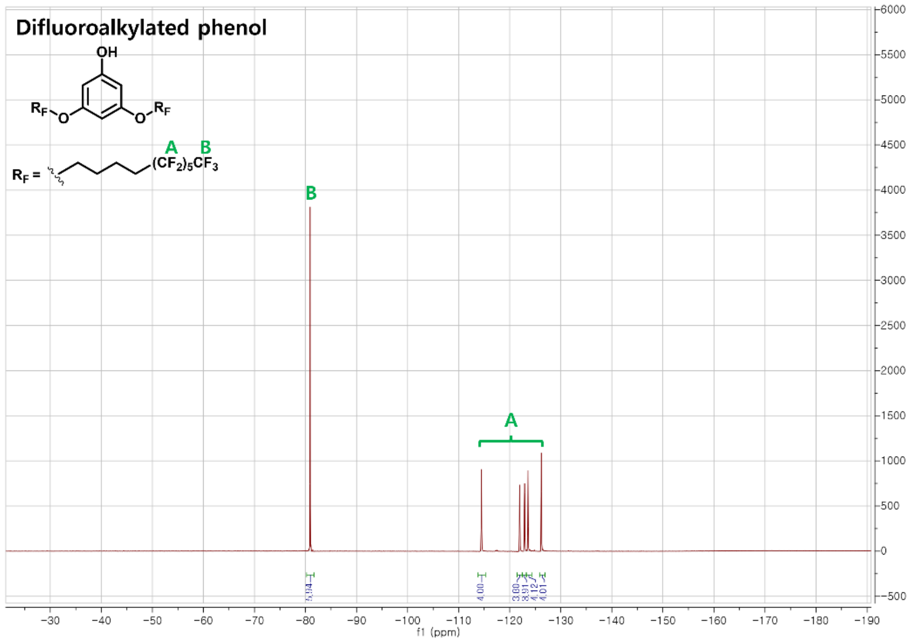

Figure S4. ^{19}F-NMR spectrum of difluoroalkylated phenol.

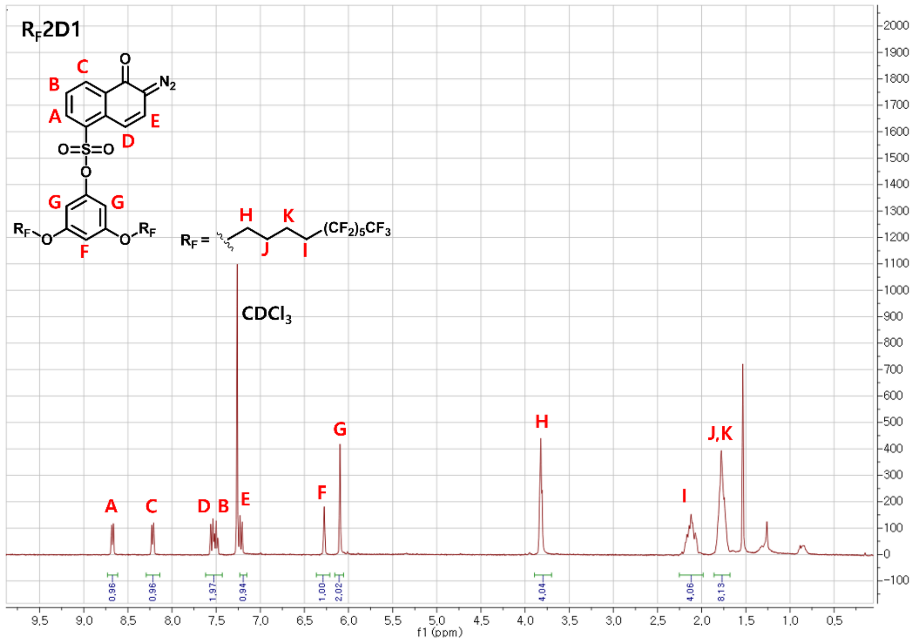

Figure S5. ^{1}H-NMR spectrum of **R$_F$2D1**.

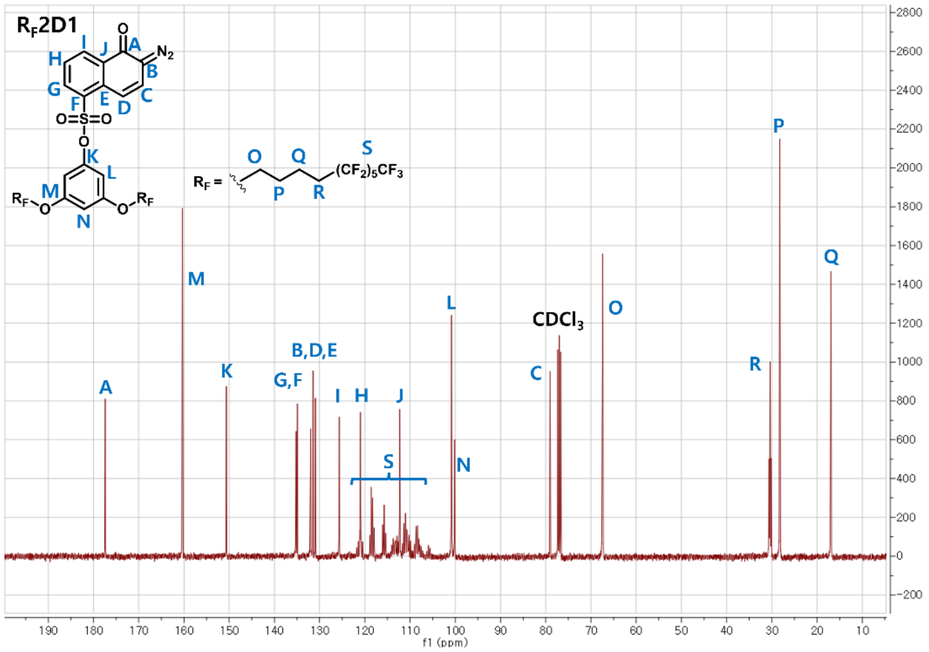

Figure S6. ^{13}C-NMR spectrum of **RF2D1**.

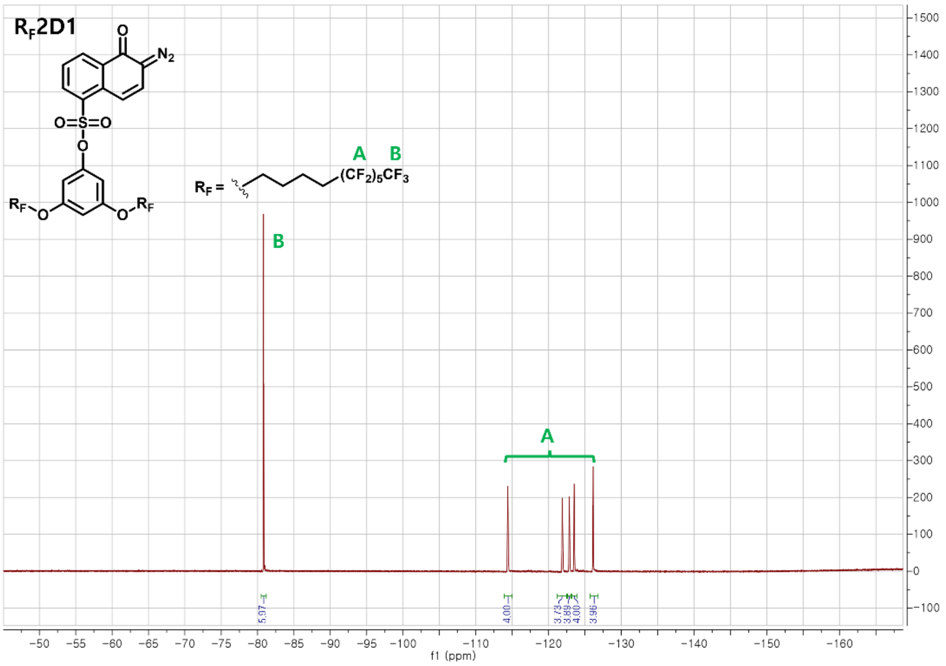

Figure S7. ^{19}F-NMR spectrum of **RF2D1**.

(a)

(b)

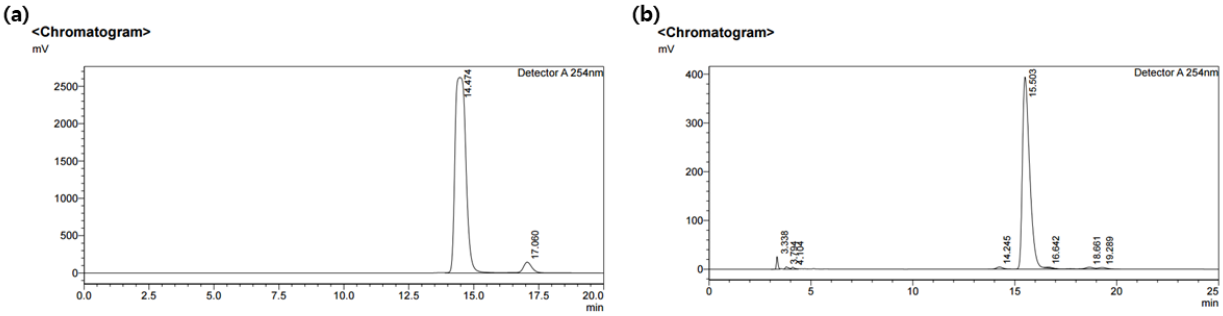

(a) <Peak Table>

Detector A 254nm

Peak#	Ret. Time	Area	Area%
1	14.474	76061716	95.516
2	17.060	3570980	4.484
Total		79632696	100.000

(b) <Peak Table>

Detector A 254nm

Peak#	Ret. Time	Area	Area%
1	3.338	138723	1.333
2	3.794	52626	0.506
3	4.104	42268	0.406
4	14.245	96925	0.932
5	15.503	9852575	94.707
6	16.642	30383	0.292
7	18.661	97165	0.934
8	19.289	92582	0.890
Total		10403247	100.000

Figure S8. HPLC chromatograms of (a) difluoroalkylated phenol and (b) **R$_F$2D1**.

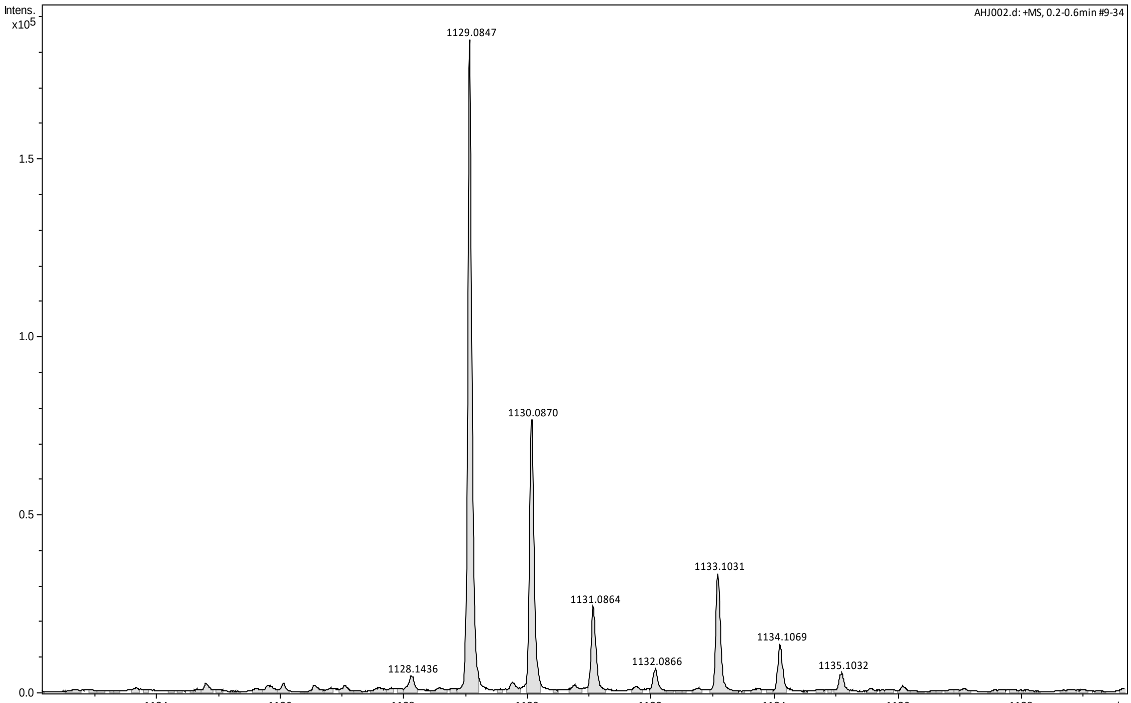

Figure S9. ESI-TOF-MS spectrum of **R$_F$2D1**.

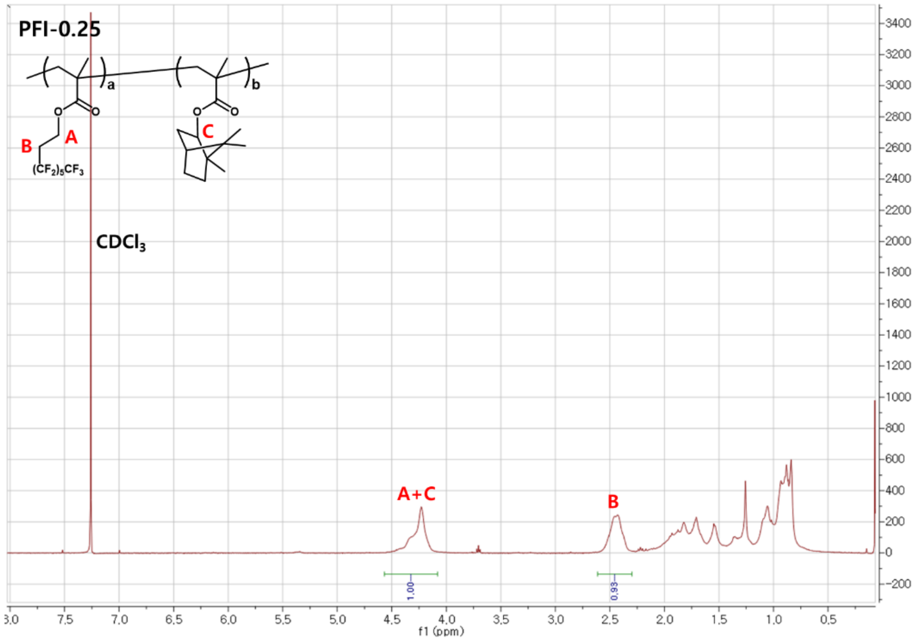

Figure S10. ^{1}H-NMR spectrum of **PFI-0.25**.

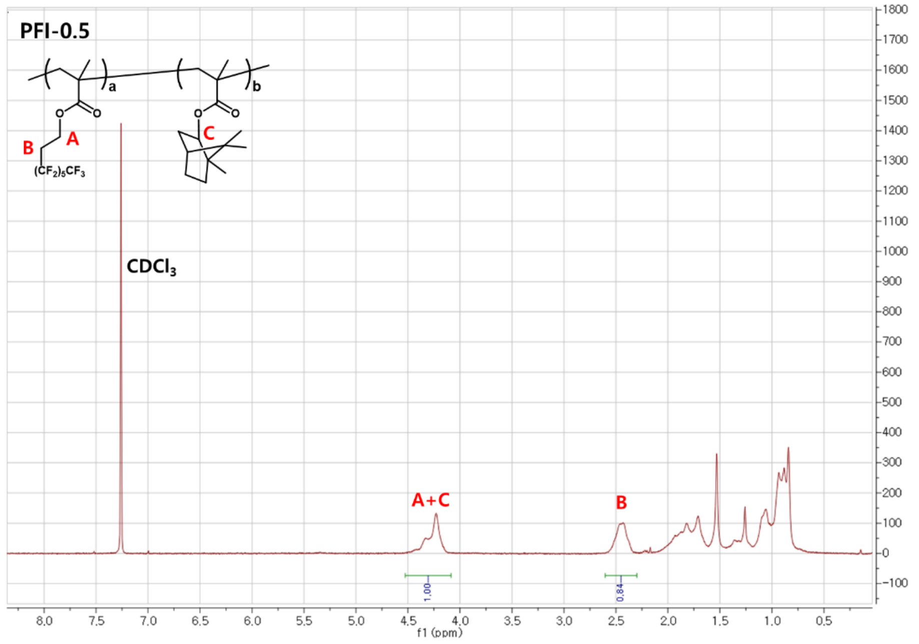

Figure S11. ^{1}H-NMR spectrum of **PFI-0.5**.

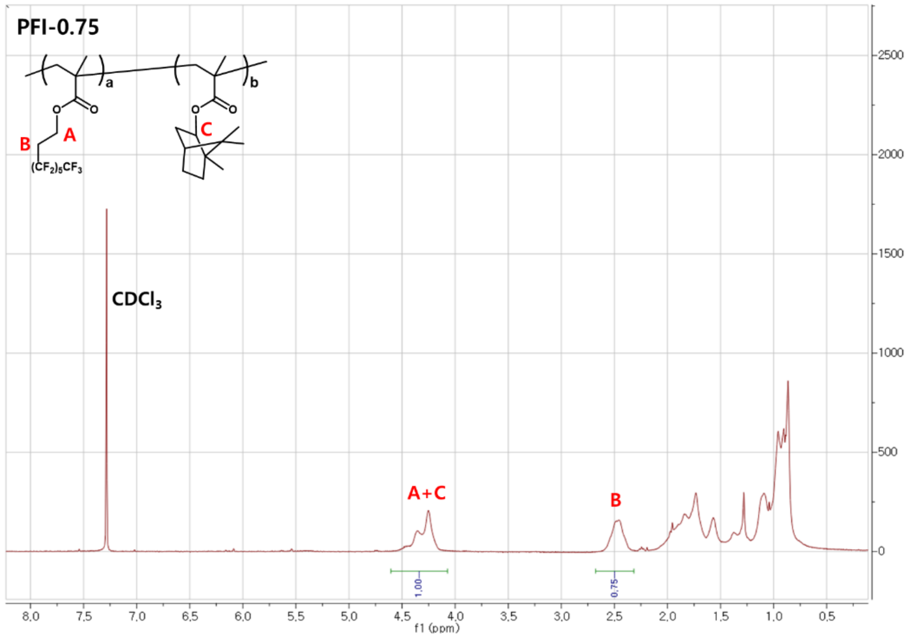

Figure S12. ^{1}H-NMR spectrum of **PFI-0.75**.

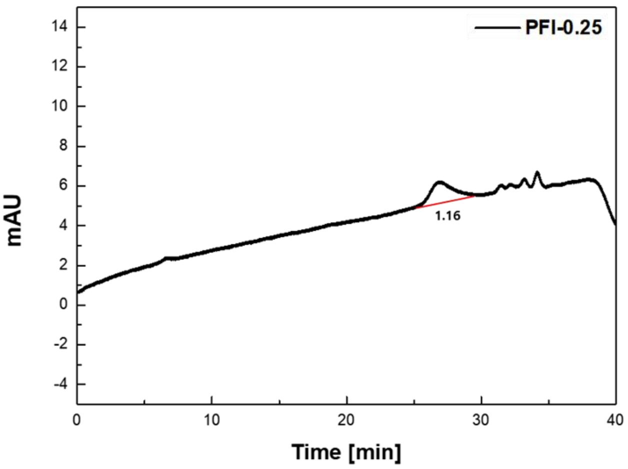

Peaks	
Peak number	1
Peak name	
**Peak Parameters**	
Ret. Time Peak Start [min]	25.51
Mw (Peak Start) [Da]	20545
Ret. Time Peak Maximum [min]	26.95
Mw (Peak Maximum) [Da]	10121
Ret. Time Peak End [min]	29.51
Mw (Peak End) [Da]	2409
Peak Area [rel. units]	1.70
% Total Peak Area	100.00
**Averages**	
Number Average Mn	8208
Weight Average Mw	9535
Z Average Mz	10716
(Z+1) Average Mz+1	11713
**Polydispersity**	
Polydispersity PD	1.16

Figure S13. GPC chromatogram of **PFI-0.25**.

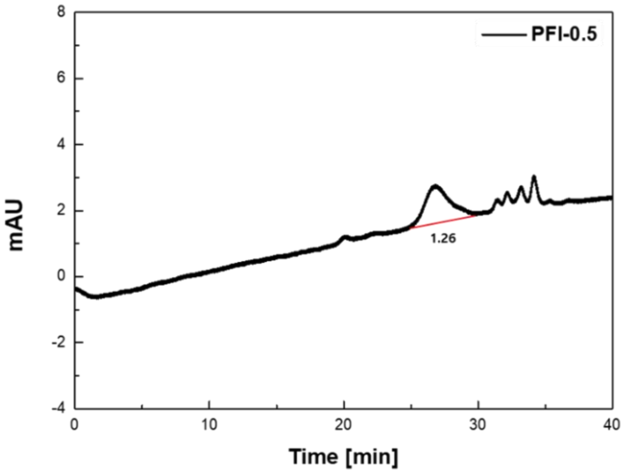

Peaks	
Peak number	1
Peak name	
**Peak Parameters**	
Ret. Time Peak Start [min]	24.70
Mw (Peak Start) [Da]	30147
Ret. Time Peak Maximum [min]	26.79
Mw (Peak Maximum) [Da]	10966
Ret. Time Peak End [min]	30.07
Mw (Peak End) [Da]	1686
Peak Area [rel. units]	2.31
% Total Peak Area	100.00
**Averages**	
Number Average Mn	8125
Weight Average Mw	10247
Z Average Mz	12232
(Z+1) Average Mz+1	14026
**Polydispersity**	
Polydispersity PD	1.26

Figure S14. GPC chromatogram of **PFI-0.5**.

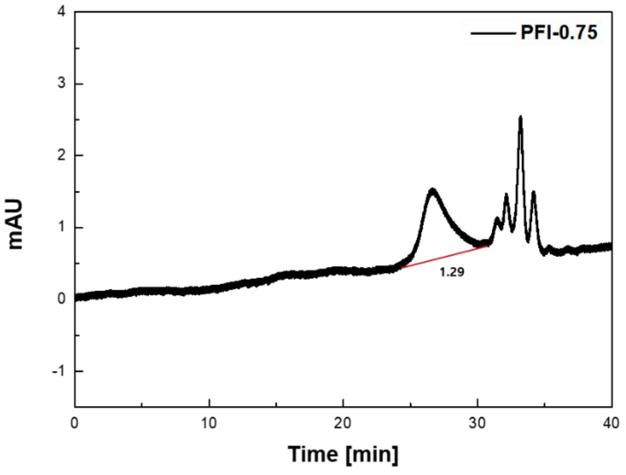

Peaks	
Peak number	1
Peak name	
**Peak Parameters**	
Ret. Time Peak Start [min]	24.35
Mw (Peak Start) [Da]	35608
Ret. Time Peak Maximum [min]	26.65
Mw (Peak Maximum) [Da]	11775
Ret. Time Peak End [min]	30.17
Mw (Peak End) [Da]	1585
Peak Area [rel. units]	2.03
% Total Peak Area	100.00
**Averages**	
Number Average Mn	8061
Weight Average Mw	10421
Z Average Mz	12363
(Z+1) Average Mz+1	13748
**Polydispersity**	
Polydispersity PD	1.29

Figure S15. GPC chromatogram of **PFI-0.75**.

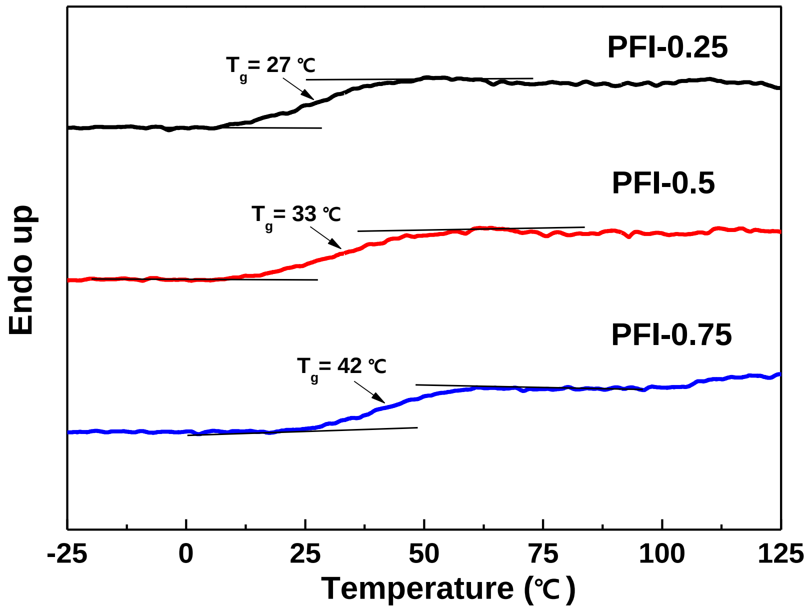

Figure S16. DSC curves of **PFI-X** (X=0.25, 0.5, 0.75).

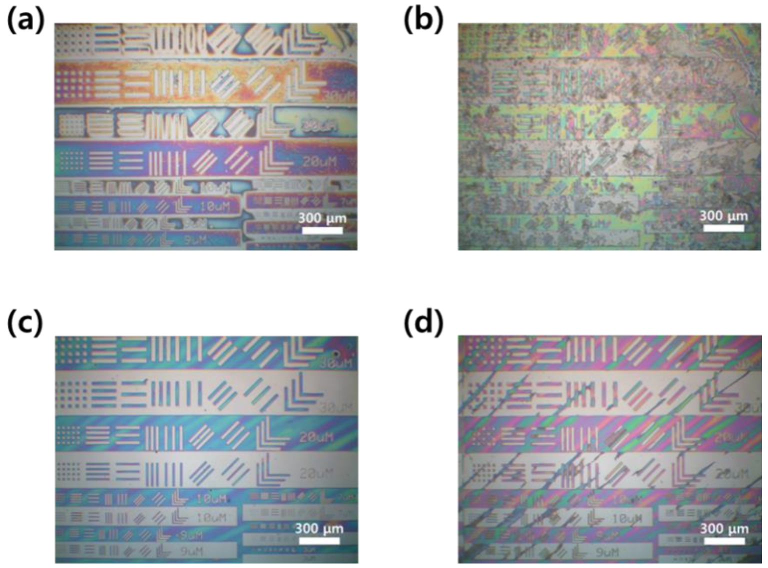

Figure S17. Optical microscope images of the films; (a) **RF2D1**, mixture of **RF2D1** and (b) **PFI-0.25**, (c) **PFI-0.5**, and (d) **PFI-0.75**.

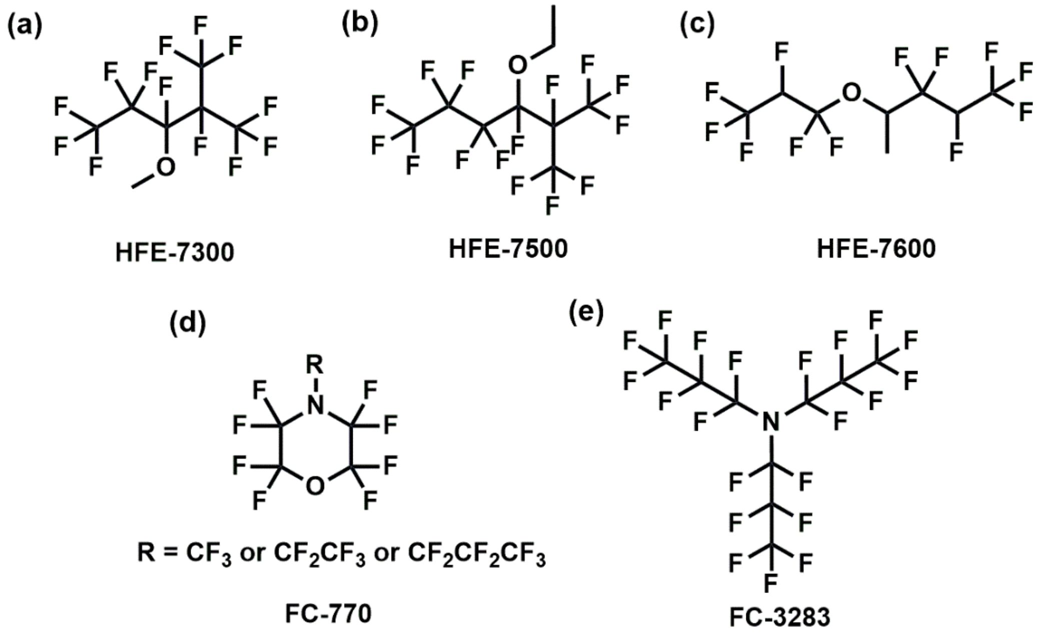

Figure S18. Chemical structures of process solvents employed in this study; (a) HFE-7300, (b) HFE-7500, (c) HFE-7600, (d) FC-770, and (e) FC-3283.

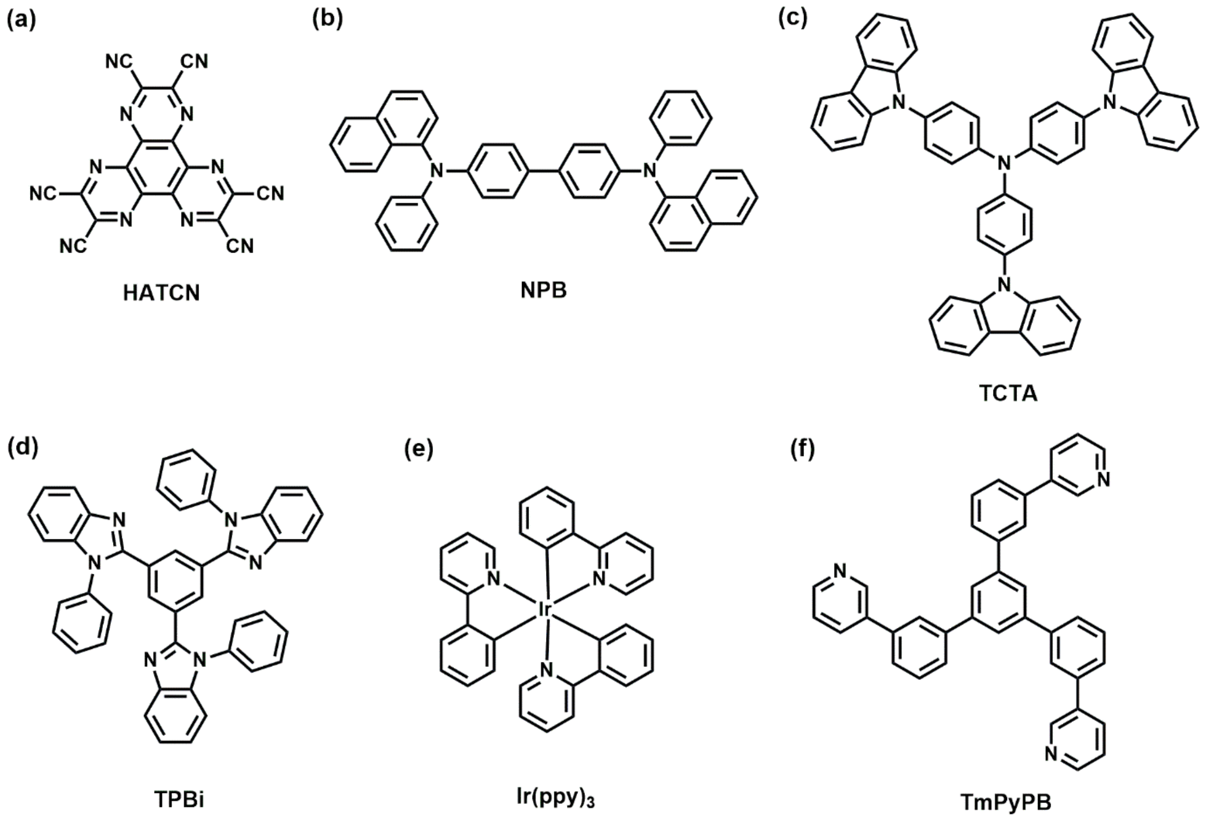

Figure S19. Chemical structures of OLED materials; (a) HATCN, (b) NPB, (c) TCTA, (d) TPBi, (e) $Ir(ppy)_3$, and (f) TmPyPB.

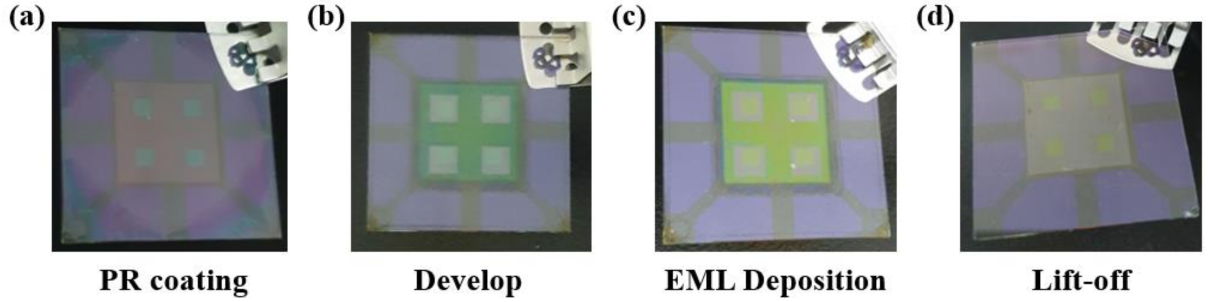

Figure S20. Photographic images of processed device.
